# Asylums of New Zealand

**Published:** 1892-12-03

**Authors:** 


### COLONIAL INSTITUTIONS.

### ASYLUMS OF NEW ZEALAND.

On December 31st, 1891, there were in New Zealand 1,849 registered insane persons distributed among seven institutions. Exclusive of Maories, this number gives a proportion of 2·92 insane per 1,000 of population, while, inclusive of Maories, the proportion is 2·74 per 1,000. In other words, there is one insane person to every 343 of the population in the former case, and one to every 365 persons in the latter. At the end of 1890 the proportion was one in 377 in New South Wales, one in 304 in Victoria, and one in 341 in England and Wales.

It may, perhaps, be thought that the proportion of insanity in our Australasian colonies is rather high, but there are various influences at work to account for it. The Inspector of Lunatic Asylums for Victoria specifies these influences as follows, and his words apply to New Zealand with equal force. Chief among the causes, he says, are:—

"(a) The facilities offered by our lunacy system for safely and cheaply disposing of weak-minded persons who have become a burden to their proper guardians. The cost of medical examination of persons suspected of being insane, as well as of the transport of lunatics and their maintenance in the asylums, is here largely borne by the State, not by municipal or district authorities as in other countries.

"(b) The poor-house system of Britain has no equivalent here, and in so far there is wanting this outlet for a large class of our asylum inmates.

"(c) The standard of mental unsoundness in use leads to many persons suffering only from old age, and harmless as regards themselves or others, being certified as insane.

"(d) The mortality among patients has been low, resulting in an accumulation of the old and incurable.

"(e) A large number of our population follow nomadic pursuits, and so are especially exposed to some of the most potent causes of mental disease."

But if the proportion is high, there is one statement in this annual report which deserves notice. Upon the completion of the new asylum at Porirua, the inspector says he will be enabled to provide sufficient cubic space for every insane person in the colony, the central block now under contract making provision for over two hundred patients of the chronic class, while the plans are so arranged that at a comparatively small cost the accommodation can be extended as the population increases. Upon this statement New Zealand is distinctly to be congratulated, although commendation must be modified by the fact that no separate accommodation appears to be provided for insane criminals, idiots, nor inebriates. The law requires that there shall be such separate buildings for inebriates, and a judge of the Supreme Court having lately ordered the release of such a patient on the ground that there was no ward in which he could be detained apart from the insane, it is possible that public opinion will compel a vote for the making of proper provision for this class.

The movement of population was briefly as follows: On the 1st of January, 1891, there were 1,797 persons in asylums, and 435 were admitted or re-admitted during the year, giving a total of 2,232 under treatment. Of these 162 were discharged cured, 57 relieved, 44 not improved, and 120 died thus leaving 1,849 under treatment on the 31st of December The average number resident during the year was 1,789·25, and the percentage of deaths of this number was only 6·71, as compared with 7·45 in Victoria, and 9·81 in England. The percentage of deaths on admissions was 27·9, and of recoveries 37·24.

The total expenditure in 1891 of all the asylums was £45,561 12s. 6d., and deducting £9,096 13s. 5d. accruing from repayments, sale of produce, and so on, the actual cost was £36,464 19s. 1d. There was a slight increase of 5s. 11½d. in the cost per head, which was £20 16s 2½d. in 1891, as compared with £20 10s. 3d. in 1890. This is accounted for by the fact that as greater vigilance is now exercised in collecting maintenance payments, there are no accumulated arrears to come in. Thus the total receipts for 1891 were 7s. 7½d. less per patient than for 1890, so that the money actually spent in 1891 was really 1s. 8d. per patient less than in 1890.

No details are furnished as to the calibre of the attendant staff, but systematic courses of instruction have been instituted at several asylums, and at two of the largest asylums the matrons are thoroughly trained professional nurses. On the whole, the insane population of New Zealand appear to be well cared for, and the asylums are administered with intelligence and efficiency.

